# A cost-effective RNA sequencing protocol for large-scale gene expression studies

**DOI:** 10.1038/srep09570

**Published:** 2015-04-01

**Authors:** Zhonggang Hou, Peng Jiang, Scott A. Swanson, Angela L. Elwell, Bao Kim S. Nguyen, Jennifer M. Bolin, Ron Stewart, James A. Thomson

**Affiliations:** 1 Morgridge Institute for Research, Madison, Wisconsin, United States of America; 2 Department of Cell and Regenerative Biology, University of Wisconsin-Madison, Madison, Wisconsin, United States of America; 3 Department of Molecular, Cellular, and Developmental Biology, University of California Santa Barbara, Santa Barbara, California, United States of America

## Abstract

RNA sequencing has increasingly become an indispensable tool for biological research. While sequencing costs have fallen dramatically in recent years, the current cost of RNA sequencing, nonetheless, remains a barrier to even more widespread adoption. Here, we present a simple RNA sequencing protocol with substantially reduced costs. This protocol uses as little as 10 ng of total RNA, allows multiplex sequencing of up to 96 samples per lane, and is strand specific. Extensive validation using human embryonic stem cells showed high consistency between technical replicates at various multiplexing levels.

RNA sequencing measures gene expression by sequencing cDNA libraries converted from mRNA and counting reads that map to each gene. Since its introduction six years ago^1^,^2^, RNA sequencing has gained popularity over microarray-based gene expression analysis. Compared to microarray analysis, RNA sequencing provides an improved dynamic range for expression level quantification and improved gene sequence information down to single base resolution^3^. RNA sequencing has also been used for gene isoform detection, gene alternative start and end mapping, and novel transcript identification^4^. RNA sequencing is an indispensable tool in biological research and will likely gain even more widespread adoption with time.

The most critical step in RNA sequencing is the construction of a cDNA library suitable for sequencing. Several protocols for this purpose have been developed^5^,^6^,^7^,^8^,^9^,^10^,^11^,^12^,^13^,^14^,^15^,^16^. The protocols can be classified into two main categories: non-stranded protocols, such as Illumina's TruSeq RNA Sample Preparation Kit in which RNA sense and antisense strand information is lost, and stranded protocols, such as Illumina's TruSeq Stranded mRNA Sample Preparation Kit in which the strand information is preserved. Non-stranded protocols generally cost less and have fewer steps compared to stranded protocols and perform well in most gene expression quantifications but lose critical information especially with regard to anti-sense transcription, which is becoming recognized as increasingly important for gene regulation^17^,^18^. Strategies to preserve transcript strand information include adaptor ligation at the RNA level^10^ or single strand cDNA level^7^, reverse transcription with primers containing one adaptor^6^, or dUTP incorporation during the second strand synthesis of cDNA^5^,^8^,^13^,^15^,^16^. Due to the extremely high percentage of ribosomal RNA in the total RNA preparation, most of the RNA sequencing protocols selectively sequence poly-A-tailed mRNA transcripts in eukaryotic cells. For a more comprehensive measurement of the whole transcriptome, ribosomal RNA depletion can be performed before library construction in place of mRNA selection.

One obstacle that prevents the wider use of RNA sequencing is the high cost of cDNA library preparation using commercially available kits. In this report, we developed a strand-specific RNA sequencing library construction protocol (LM-Seq: Ligation Mediated RNA sequencing) that dramatically reduces the cost of sample preparation. Reagents used in this protocol are fully disclosed and widely available. The whole protocol is highly streamlined and a single researcher can process up to 192 samples in two days by hand. We also reduced sequencing costs by designing indexes to allow multiplex sequencing up to 96 samples per lane. Using this protocol, we sequenced up to 95 technical replicates of mRNAs from human embryonic stem cells and found this protocol to produce highly consistent results between technical replicates at various multiplexing levels.

## Results

### Characterization of RNA sequencing library prepared by LM-Seq

**Figure 1:**
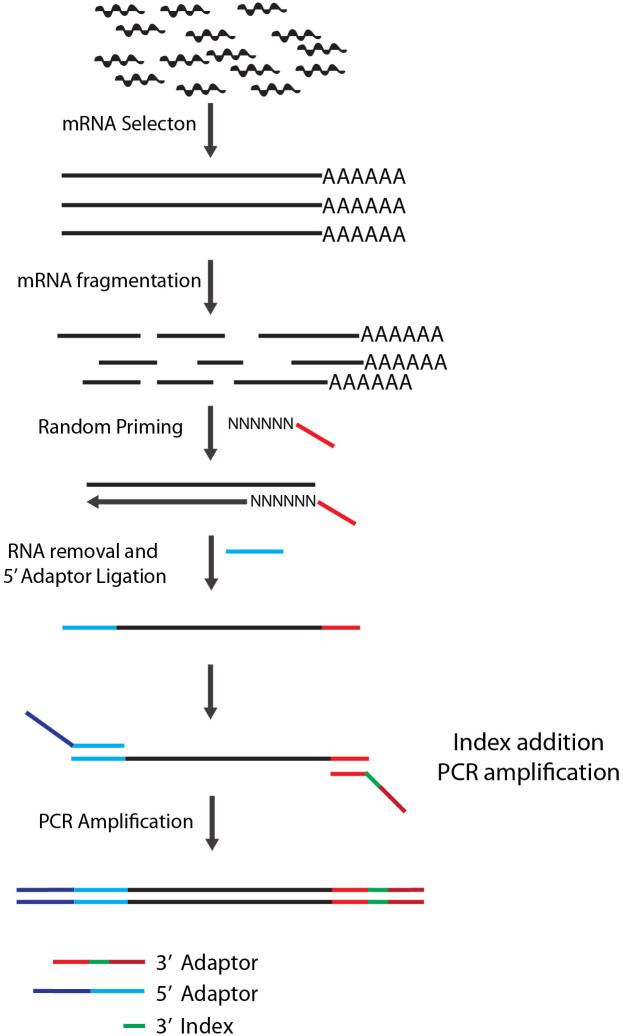
Diagram of LM-Seq sample preparation protocol. Poly-A-tailed mRNA is isolated from total RNA using oligo-dT beads. Purified mRNA is then fragmented with heat in fragmentation buffer. First strand cDNA is then synthesized using random hexamer oligos containing partial Illumina 3′ adaptor sequence. After RNA removal, a modified oligo containing partial Illumina's 5′ adaptor is then ligated to the 5′ of the single stranded cDNA. The library is then amplified by PCR using oligos that contain full Illumina adaptor sequences and our in-house index sequences.

Figure 1 illustrates the general steps of Ligation Mediated RNA sequencing library prep, or LM-Seq. We first purified mRNA from total RNA using oligo-dT beads. Purified mRNA was then fragmented by heat in reverse transcriptase buffer and reverse-transcribed with a random hexamer oligonucleotide. To streamline this protocol and reduce costs, we incorporated a partial sequence from Illumina's 3′ adaptor to this oligo, which would serve as an annealing site during the final PCR amplification stage when the full 3′ adaptor is added. We then removed the RNA and ligated a modified oligo containing partial sequence from Illumina's 5′ adaptor to the single stranded cDNA. This oligo has a 5′ phosphate to allow ligation with the cDNA using T4 RNA ligase and 3′ di-deoxycytosine to prevent self-ligation. During the final PCR amplification step, full Illumina 5′ and 3′ adaptors were introduced via PCR. To allow for multiplexing, we incorporated index sequences within the 3′ adaptor, which has the index sequencing primer annealing site for Illumina's Small RNA sequencing primer.

**Figure 2:**
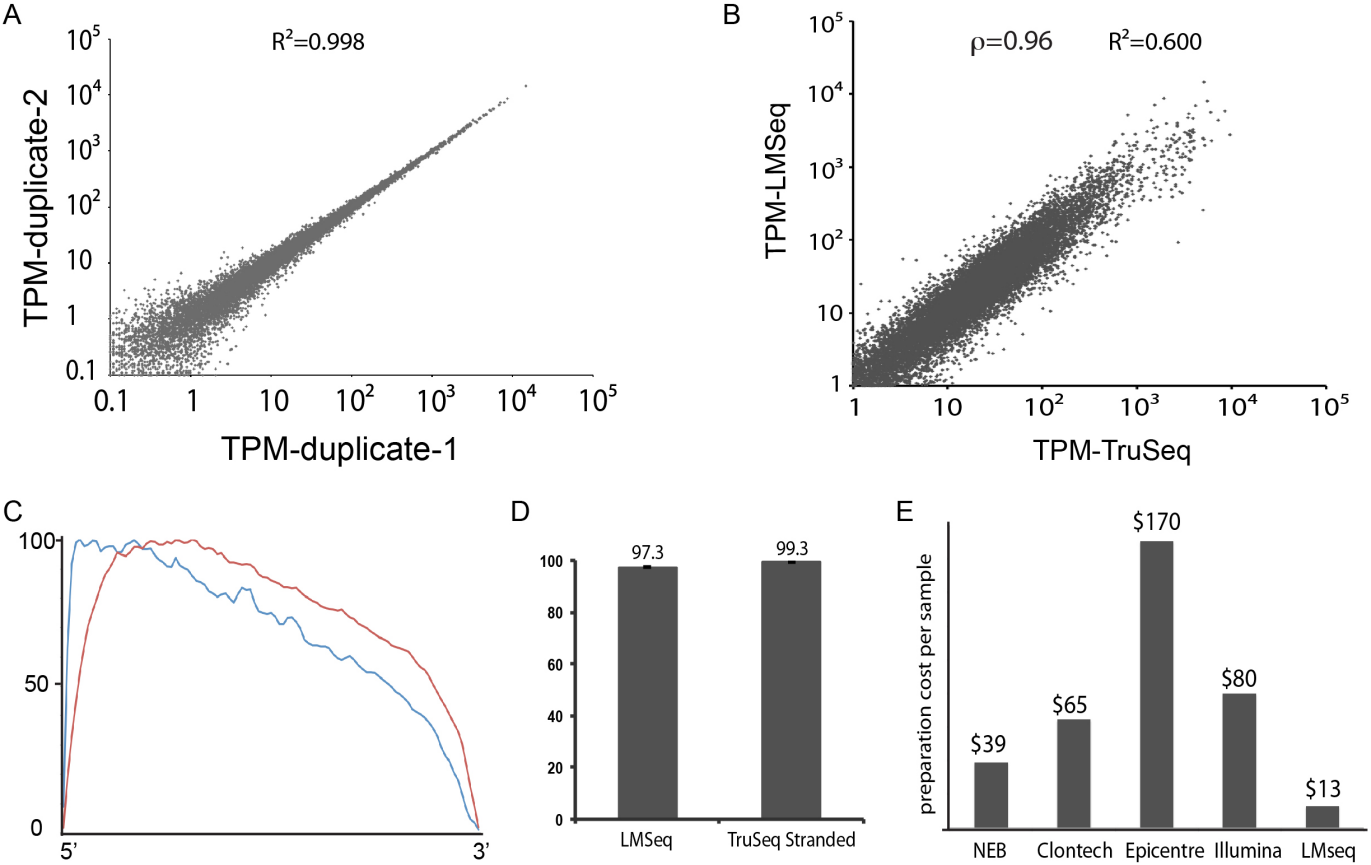
Performance and cost of LM-seq protocol. (A). Scatter plot of TPM values of two technical replicates of LM-Seq of hES cells using 100 ng total RNA as starting material. Pearson correlation is reported on top of the plot. (B). Scatter plot of TPM values of LM-Seq and TruSeq of hES cells using 100 ng total RNA as starting material. Spearman's rank correlation (ρ) and Pearson correlation are reported on top of the plot. (C). Average reads distribution across all transcripts. Blue: LM-Seq, Red: TruSeq. (D). The comparison of the percentage of reads that map to the correct strands of the human transcriptome between data generated by LM-Seq and TruSeq Stranded mRNA-Seq protocol (error bar: +/−standard deviation). (E). Comparison of preparation cost per sample between commercially available kits (NEBNext Ultra Directional RNA Library Prep Kit from NEB, ScriptSeq Complete Kit from Epicentre, SMARTer Stranded RNA-seq Kit from Clontech, TruSeq Stranded mRNA Kit from Illumina) and LM-Seq.

To test the robustness of this protocol, we prepared cDNA libraries from total RNA isolated from human embryonic stem cells. We started with 100 ng of total RNA for each sample. As shown in Figure 2A, technical duplicates of the same sample have a very high correlation (Pearson R^2^ = 0.998), indicating the protocol is very consistent. The Spearman's rank correlation between the same hES cell sample sequenced with LM-Seq and Illumina's TruSeq is 0.96 (Fig. 2B), indicating a high correlation between the two protocols. However, since these two protocols use different chemistry for library construction, the absolute value for each gene cannot be compared, which is indicated by the low Pearson correlation between data generated by these two protocols (Fig. 2B). The read coverage of LM-Seq showed a 5′ bias (Fig. 2C), similar to Illumina's TruSeq mRNA protocol (Fig. 2C). This is likely due to the degree of RNA fragmentation before reverse transcription (Supplemental Fig. 1).

One important feature of this protocol is that it allows for strand-specific RNA sequencing. When dealing with transcriptome datasets, the ability to differentiate sense and anti-sense strand transcripts is very important. Within all reads that map to the human transcriptome, we calculated the percentage of reads that map to the correct strand. As shown in Figure 2D, a very high portion of reads (>97%) generated by LM-Seq map to the correct strand (sense strand). This number is similar to percentage of reads mapping to the correct strand using TruSeq Stranded mRNA protocol (Fig. 2D and Table 1) or other previously reported stranded RNA sequencing protocols compared by Levin et al (LM-Seq is most similar to NNSR with no ActD)^19^.

Our major motivation for developing LM-Seq was to reduce the cost of library preparation. We calculated the reagent and consumable cost of LM-Seq (Supplemental Table 1) and compared it with popular commercially available strand-specific RNA sequencing library construction kits. LM-Seq cost significantly less (3 to 13 fold) than those commercially available kits (Fig. 2E).

### LM-Seq can start with as little as 10 ng total RNA

**Figure 3:**
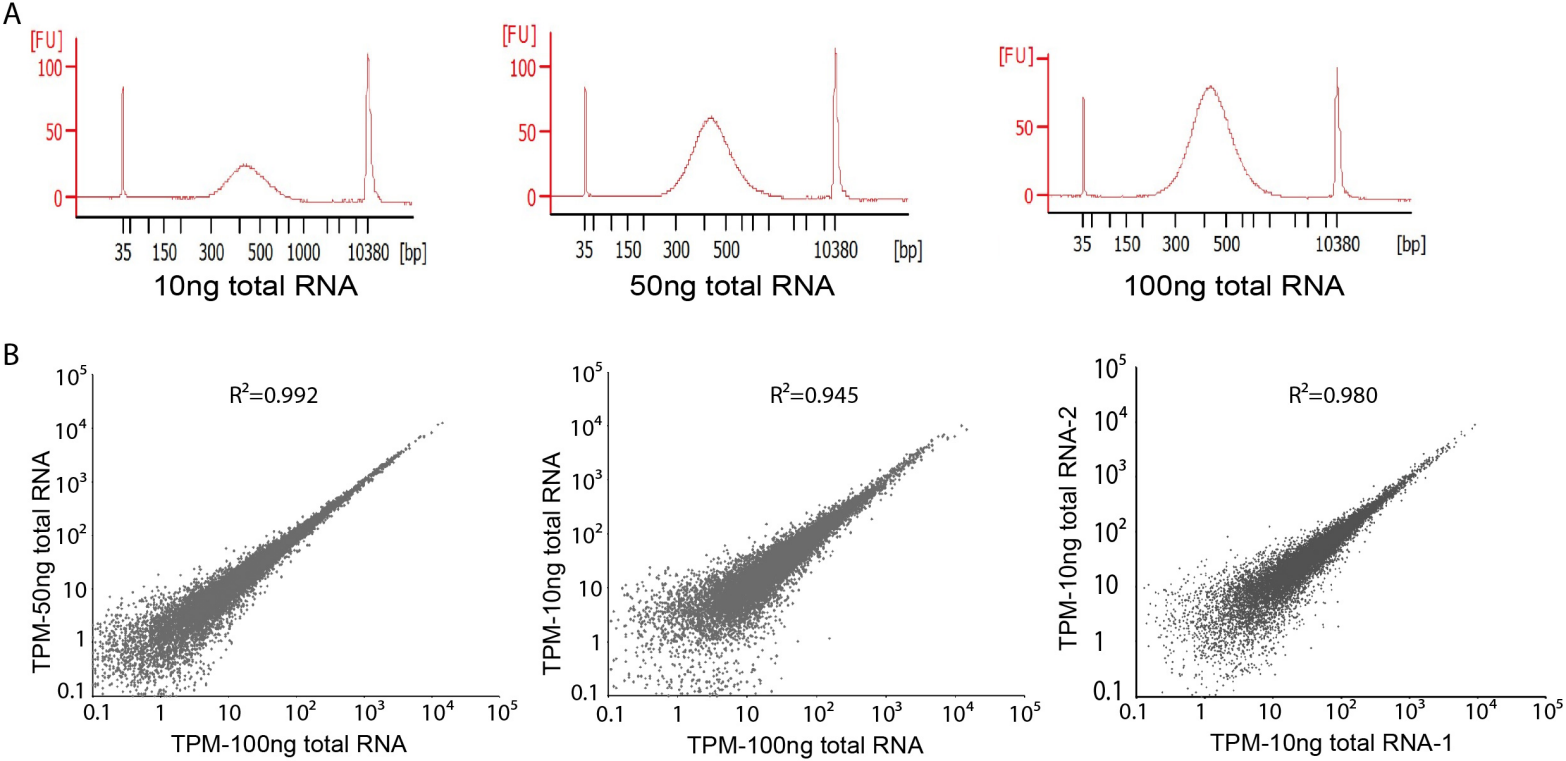
Performance of LM-Seq with different amount of starting material. (A). Bioanalyzer electropherograms of final library starting from 10 ng, 50 ng, and 100 ng of total RNA. (B). Scatter plots of TPM between samples starting with various amount of total RNA. Pearson correlation is reported on top of the plots.

During large-scale screening or high throughput experiments, the amount of total RNA for sequencing library construction might be limited. Although the 100 ng total RNA we used above is considered a very low amount of starting material, we wanted to test if our protocol could be effective with even lower amounts. We tested both 50 ng and 10 ng total RNA as starting material. The overall size distribution of the final cDNA library starting from 50 ng or 10 ng total RNA was very similar to that made from 100 ng total RNA (Fig. 3A). We then compared the sequencing results of those cDNA libraries. Gene expression quantification from both libraries showed high correlation with the one generated from 100 ng total RNA (Pearson R^2^ = 0.992 and R^2^ = 0.945, respectively) (Fig. 3B). Libraries generated from 10 ng total RNA also showed high correlation between technical replicates (Fig. 3B). We did observe a lower correlation between technical duplicates from 10 ng and 100 ng samples (Fig. 3B), and that might be in part due to the loss of complexity when starting material is limited.

### Highly multiplexed RNA sequencing using LM-Seq

**Figure 4:**
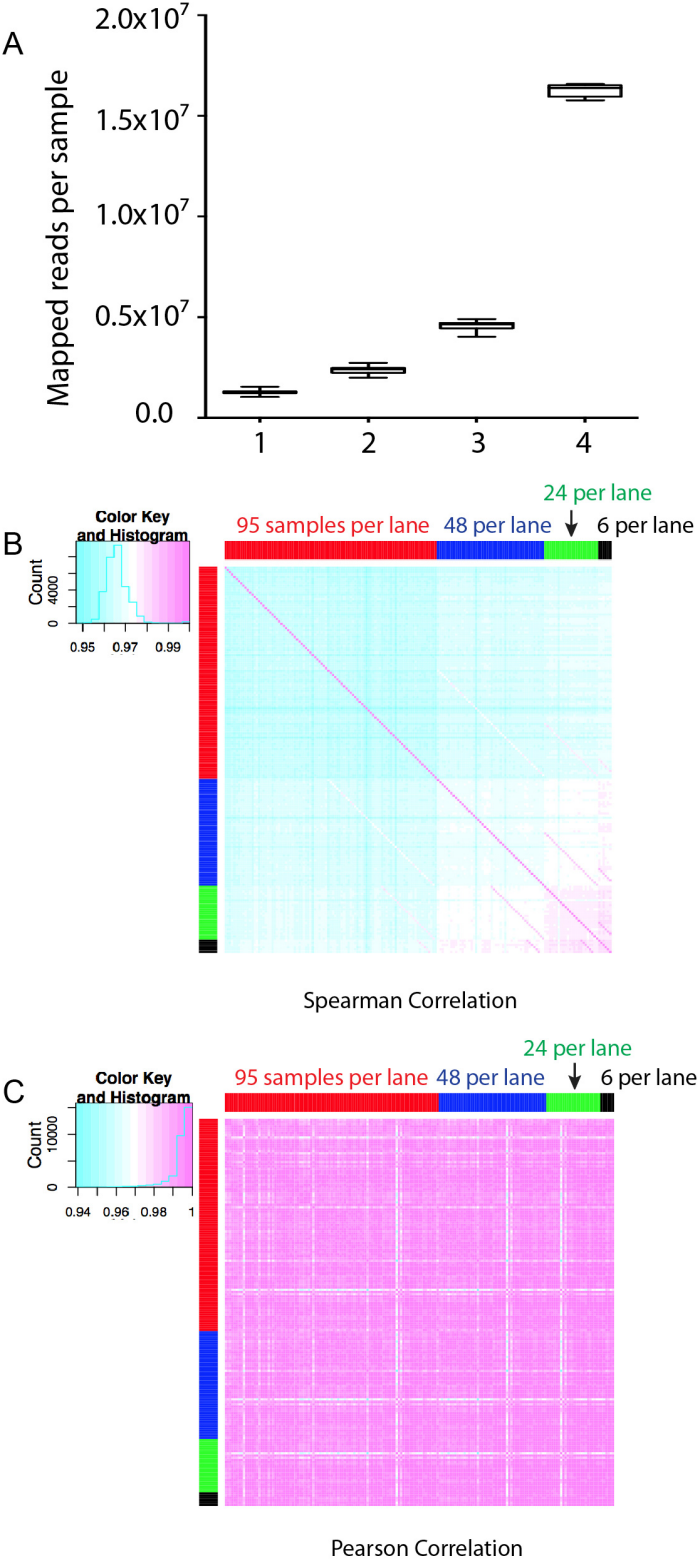
Performance of LM-Seq with highly multiplexed samples per lane. (A). Box-Whisker plot showing the distribution of the number of mapped reads among indexed samples in lanes with 95 samples (lane 1), 48 samples (lane 2), 24 samples (lane 3), and 6 samples (lane 4). (B). Pairwise Spearman correlation of samples sequenced with different multiplex levels per lane. (C). Pairwise Pearson correlation of samples sequenced with different multiplex levels per lane.

With the current HiSeq2500 from Illumina, we routinely get >150 M reads per lane of sequencing, which is much more than needed for standard gene expression analysis. So next we evaluated the performance of LM-Seq with highly multiplexed samples. To accomplish that, we designed a set of 10 nt DNA indexes with 5 nt editing distance between any two indexes (Supplemental Table 2). Two major challenges often seen with high level multiplexing are the uneven distribution of reads between samples in the same lane and inconsistent gene expression quantification due to the lowered sequencing depth. To systematically investigate those challenges, we prepared 95 independent cDNA libraries with different indexes from the same total RNA. We then sequenced those samples with different levels of multiplicity (6, 24, 48, and 95 samples per lane). We first looked at the reads distribution. As shown in Figure 4A, the distribution of reads across all samples is very uniform. Even with 95 samples per lane, there is only a less than 2-fold difference between the sample with the highest number of reads and the sample with the lowest. We then looked at the effect of read depth at gene quantification. We performed both pairwise Spearman's rank correlation and pairwise Pearson correlation analysis (Fig. 4B and C). In this case, Pearson correlations were typically higher. The difference is very obvious between samples from 95 or 48 per lane and less so for samples from 24 or 6 per lane. This suggests that with lower sequencing depth, highly expressed genes are probably not affected much while genes with a low expression level start to show inconsistency since Pearson correlation is based on the actual value and thus is more affected by highly expressed genes while the Spearman correlation is based on the rank of each gene. Further studies are required to determine the proper sequencing depth for different transcriptome studies.

## Discussion

We developed LM-Seq as a cost-effective strand-specific RNA sequencing protocol to enable large-scale comparative gene expression analysis. This protocol is highly streamlined, with minimal hands on time. It is fully compatible with multi-channel pipetting to allow parallel processing of a large number of samples. A single person can process up to 192 samples within two days. The most time consuming step in this protocol is the ligation of the partial Illumina's 5′ adaptor. If preferred, this ligation time can be shortened to as little as one hour with minimal affect on gene quantification (Supplemental Figure 2). However, we did observe a lowered final library yield with shortened ligation time and also a decreased percentage of non-duplicated reads (Table 1). Whenever the highest library quality is needed, overnight ligation is still recommended.

Data generated by LM-Seq showed high correlation with those generated by Illumina's TruSeq (Fig. 2B). However, since these two protocols use different chemistries for library construction, it is best to compare data generated using the same protocol. LM-Seq can also be adapted for paired-end sequencing by replacing 3′ adaptor sequence with that from the Illumina's TruSeq adaptors.

LM-Seq selectively sequences poly-A-tailed mRNA transcripts. Information about RNA transcripts that are not poly-A-tailed, such as some forms of non-coding RNAs, will be lost. If information for non-poly-A-tailed transcripts is desired, the mRNA selection step in LM-Seq can be replaced with a ribosomal RNA reduction step using commercially available kits, such as Ribo-Zero rRNA removal kit from Epicentre.

Read coverage using LM-Seq has a slight bias towards the 5′ end of the transcript (Fig. 2C), which is related to the level of RNA fragmentation before reverse transcription (Supplemental Figure 1). This is partly due to the fact that the Illumina platform only sequences the first 50–100 nt from the 5′ end of the cDNA during a single end read run. In order to get read coverage at the 3′ end of the transcript, the length of fragmented RNA needs to approach the length of reads, which is sometimes not practical. The average insert length in LM-Seq is around 300 bp, while the average insert length in TruSeq is around 160–170 bp, which explains the slightly higher 5′ bias for LM-Seq. Indeed, when we increased either the fragmentation time or temperature to shorten the RNA length after fragmentation, the 5′ bias is reduced (Supplemental Figure 1). However, for unclear reasons, libraries generated from shorter RNA fragments showed a higher percentage of duplicated reads (Table 1), which indicated a loss of read complexity. For comparative gene expression analysis, a slight 5′ bias in read coverage is not a concern since this bias is small and uniform across all samples. For applications that require a more uniform coverage, a longer RNA fragmentation time and a longer sequencing read length might be needed for LM-Seq.

With the ever-increasing data output per lane of a flowcell on the Illumina platform, the ability to multiplex becomes critical for any RNA sequencing protocol. We designed a set of 96 indexes for LM-Seq for this purpose. These indexes have a 5 nt editing distance between any two indexes to allow unambiguous differentiation between samples with different indexes. With an increased number of samples per lane, the sequencing depth per sample becomes a concern, especially for complex transcriptomes like that of human. Our data suggested that genes with low expression values suffer more than genes with high expression values when sequencing depth is limited. For pilot screen experiments where large changes in gene expression are expected and/or tracking expression patterns of high or moderate expressers is the goal, multiplexing with up to 96 samples per lane for human is likely adequate. Also, for species such as yeast that have a less complex transcriptome, one can probably multiplex even more without significant loss of sensitivity.

## Methods

### Cell culture and RNA isolation

Human embryonic stem cell line H1 was cultured on Matrigel coated plates in E8 medium as previously described^20^. For RNA purification, cells were lysed directly on plate with RLT lysis buffer (Qiagen) and total RNA was purified with RNeasy Mini Kit (Qiagen).

### RNA sequencing library prep with LM-Seq

A detailed step-by-step protocol can be found in the supplemental file. Briefly, mRNA is isolated from purified 100 ng total RNA using oligo-dT beads (NEB). Isolated mRNA is fragmented in reverse transcription buffer with heat and then reverse-transcribed with SmartScribe reverse transcriptase (Clontech) using a random hexamer oligo (HZG883: CCTTGGCACCCGAGAATTCCANNNNNN). After reverse transcription, RNA is removed by RNaseA and RNaseH treatment. A partial Illumina 5′ adaptor (HZG885:/5phos/AGATCGGAAGAGCGTCGTGTAGGGAAAGAGTGTddC) is then ligated to the single stranded cDNA using T4 RNA ligase 1 (NEB) and incubated overnight at 22°C. After purification, ligated cDNA is amplified by 18 cycles of PCR using oligos that contain full Illumina adaptors (LC056: AATGATACGGCGACCACCGAGATCTACACTCTTTCCCTACACGACGCTCTTCCGATCT and Index primer: CAAGCAGAAGACGGCATACGAGATnnnnnnnnnnGTGACTGGAGTTCCTTGGCACCCGAGAATTCCA, nnnnnnnnnn indicates index nucleotides). For testing of a low amount of input samples, reverse transcription is done with either SuperScriptIII (Life Technologies) or SmartScribe, and the cycle of PCR is increased to 19 cycles for 50 ng total RNA and 20 cycles for 10 ng total RNA.

### Sequencing of cDNA library and data processing

Indexed cDNA libraries are pooled and sequenced on an Illumina HiSeq2500 with a single 51 bp read and a 10 bp index read. FASTQ files were generated by CASAVA (v1.8.2). Reads were mapped to the human transcriptome (RefGene v1.1.17) using Bowtie^21^ (v0.12.8) allowing two mismatches and a maximum of 20 multiple hits. The gene expression values (Transcript per Million Reads or TPM) were calculated by RSEM^22^ (v1.2.3).

## Author Contributions

Z.H., S.S., R.S. and J.A.T. designed research; Z.H., A.E., B.K.N. and J.B. performed research; Z.H., P.J., R.S. and J.A.T. analyzed data; and Z.H. and J.A.T. wrote the paper.

## Supplementary Material

Supplementary Information Supplementary Information

## Figures and Tables

**Table 1:** Statistics of libraries generated by TruSeq or LM-Seq

Samples	Multiplex per lane	Total reads	Percent of non-duplicated reads*	Number of reads mapped to RefSeq	Percent of reads mapping to RefSeq	Number of genes withTPM > 1	Percent of mapped reads mapping on expected sense/antisense strand
TruSeq	12	12426491	62.4%	10929050	88%	13000	50.6%
TruSeq-Stranded mRNA Seq-1	6	28515378	33.5%	24787808	87%	12151	99.4%
TruSeq-Stranded mRNA Seq-1	94	2179618	72.3%	1910603	88%	12899	99.4%
LM-Seq-1	24	6417881	62.0%	5119315	80%	12484	97.2%
LM-Seq-2	24	8786110	53.6%	5985757	68%	12784	98.2%
LM-Seq-10 ng total RNA	24	5673905	21.1%	3770987	66%	12421	98.6%
LM-Seq-85°C 11 min	24	8391832	47.8%	5601727	68%	12752	98.2%
LM-Seq-94°C 6 min	24	8273910	27.0%	5545433	67%	12558	98.2%
LM-Seq-1 hr ligation	24	6116079	35.5%	4172809	68%	12624	98.0%
LM-Seq-3 hr ligation	24	6613601	42.7%	4381615	66%	12668	98.2%

*: As reported by FastQC version 0.11.2.
