# Supplementary material for: A cost-effective RNA sequencing protocol for large-scale gene expression studies
Source: Sci Rep. 2015 Apr 1;5:9570. doi: 10.1038/srep09570 (PMC4381617; doi:10.1038/srep09570)
Supplement: Supplementary Information [file srep09570-s1.pdf]

Supplemental Information

Title: A cost effective RNA sequencing protocol for large-scale gene expression studies

Running title: **A cost effective RNA sequencing protocol**

Authors:

Zhonggang Hou (Co-corresponding author)
Morgridge Institute for Research
Madison, Wisconsin 53715, United States of America

Peng Jiang
Morgridge Institute for Research
Madison, Wisconsin 53715, United States of America

Scott A. Swanson
Morgridge Institute for Research
Madison, Wisconsin 53715, United States of America

Angela L. Elwell
Morgridge Institute for Research
Madison, Wisconsin 53715, United States of America

Bao Kim S. Nguyen
Morgridge Institute for Research
Madison, Wisconsin 53715, United States of America

Jennifer M. Bolin
Morgridge Institute for Research
Madison, Wisconsin 53715, United States of America

Ron Stewart
Morgridge Institute for Research
Madison, Wisconsin 53715, United States of America

James A. Thomson (Co-corresponding author)
Morgridge Institute for Research
Madison, Wisconsin 53715, United States of America

Department of Cell and Regenerative Biology, University of Wisconsin-Madison,
Madison, Wisconsin 53706, United States of America

Department of Molecular, Cellular, and Developmental Biology, University of California Santa Barbara
Santa Barbara, California, United States of America

Supplemental Methods

Detailed LM-Seq library prep protocol

1. mRNA Isolation (NEB Next Poly A+ Isolation Kit, NEB #E7490S/L)-Follow protocol with the following exceptions:

a. Use 15µl beads per sample. Wash twice with 100µl 2X binding buffer, then resuspend in 50µl 2X binding buffer. If processing multiple samples at the same time, wash beads in bulk using 15ml conical tubes. Dilute 100ng total RNA in water to make final volume 50µl. Then add 50µl of resuspended beads.

b. After mRNA binding, perform 1 wash instead of the recommended 2 washes.

c. For final elution, use Elute, Prime, and Fragment buffer below and proceed to 2a (do not do final elution step in NEB Next Protocol)

2. Run reverse transcription reactions (Clontech SmartScribe kit):

a. Elute, Prime, and Fragment buffer

	One reaction
H2O	5µl
5x 1st strand buffer (SmartScribe buffer)	2.5µl
3' AD with random hexamer (20µM)- HZG883	0.75µl

Add 8.25 µl to beads from each mRNA isolation, incubate at 85°C for 7 min, then cool on ice. Put on magnet and transfer 6.5 µl supernatant to step 2b.

b. cDNA synthesis

	One reaction
H2O	1µl
DTT (100mM)	0.25µl
dNTP mix (10mM each)	1µl
RNase Inhibitor	0.25µl
SmartScribe Reverse Transcriptase (100U)	1µl
Eluted mRNA from 2a	6.5µl
total	10µl

Incubate reaction at 23°C for 10min then 42°C for 30min. Then cool down reaction to RT.

c. Removing RNA

	One reaction
RNase H (2U/µl)	0.5µl
RNase A (1µg/µl)	0.5µl
cDNA from 2b	10µl
Total	11µl

Incubate reaction at 37°C for 15min then 95°C for 5min.

3. Purify cDNA with AMpure XP beads.

a. Add 20µl AMpure XP beads to each reaction. Mix well. Incubate at RT for 15min.

b. Discard supernatant. Wash beads twice with 200µl 80% EtOH. Let the beads air dry for 5 to 10min. Beads are dry when cracks appear in the pellet.
c. Elute in 12µl EB buffer
d. Put on magnet and transfer 10µl to step 4.

4. Adaptor Ligation and PCR

a. Adaptor Ligation (NEB) (NOTE: PEG8000 is very viscous):

	One reaction
10X RNA ligase buffer	3µl
PEG8000 (50%)	9.5µl
ATP (10mM)	3µl
5' AD Oligo (20µM)- HZG885	1.5µl
T4 RNA ligase I (10000U/ml)	3µl
Eluted cDNA from step 3	10µl
Total	30µl total

Incubate at 22°C for 12 to 16 hours.
b. Stop Ligation by adding 20µl of 10 mM Tris-HCl pH 8.0, 15 mM EDTA to each reaction
c. Double AMpure XP Beads Cleanup (NOTE: during final elution, beads do not pellet to magnet as well as they normally do):

i. 1st AMpure XP Cleanup (1.8 beads:1 rxn ratio):
i1. Add 90µl Ampure XP beads to each reaction. Mix well. Incubate at RT for 15min.
i2. Discard supernatant. Wash beads twice with 200µl 80% EtOH. Let the beads air dry for 5 to 10min. Beads are dry when cracks appear in the pellet.
i3. Elute in 22µl EB buffer.
i4. Put on magnet and transfer 20µl to a fresh tube.

ii. 2nd AMpure XP Cleanup (1.8 beads:1 rxn ratio):
ii1. Add 36µl Ampure XP beads to each reaction. Mix well. Incubate at RT for 15min.
ii2. Discard supernatant. Wash beads twice with 200µl 80% EtOH. Let the beads air dry for 5 to 10min. Beads are dry when cracks appear in the pellet.
ii3. Elute in 22µl EB buffer.
ii4. Put on magnet and transfer 20µl to step 4d.

d. PCR (Epicentre's FailSafe 2x Master Mix):

	One reaction
H2O	8.5µl
5' Adaptor Primer LC056 (20µM)	0.5µl
FailSafe PCR PreMix **E**	30µl
FailSafe PCR Enzyme	0.6µl
3' Index Primer (20µM)*	0.5µl
Eluted cDNA from step 4c	20µl
Total	60.1µl

*: If multiplexing, use a primer with unique index sequence for each sample to be run in the same lane.
Run the following program:

95°C for 1 min
95°C for 10 sec ⎫
55°C for 10 sec ⎬ 18 cycles
68°C for 3 min ⎭
68°C for 7 min

e. Purify with AMpure XP beads using 1 beads:1 rxn ratio
i. Add 60ul AMpure beads to each reaction. Mix well. Incubate at RT for 15min.
ii. Discard supernatant. Wash beads twice with 200µl 80% EtOH. Let the beads air dry for 5 to 10min. Beads are dry when cracks appear in the pellet.
iii. Elute in 22µl EB buffer.
iv. Put on magnet and transfer 20µl to a fresh tube.

5. Quantify DNA concentration with PicoGreen Assay (Life Technologies) or Qubit's dsDNA HS Assay.

6. Pool samples. Skip this step if indexing is not required.

7. Purify with AMpure XP beads using 1 beads:1 rxn ratio; Elute in 27µl EB if pooling 96 or 48 samples and transfer 25µl to a fresh tube. Elute in 14µl EB if pooling less than 48 samples and transfer 12µl to a fresh tube.

8. Quantify pooled DNA with Qubit's dsDNA HS Assay and run an Agilent High Sensitivity DNA chip on the BioAnalyzer to check on the quality and the average size of the final library.

Supplemental Figures

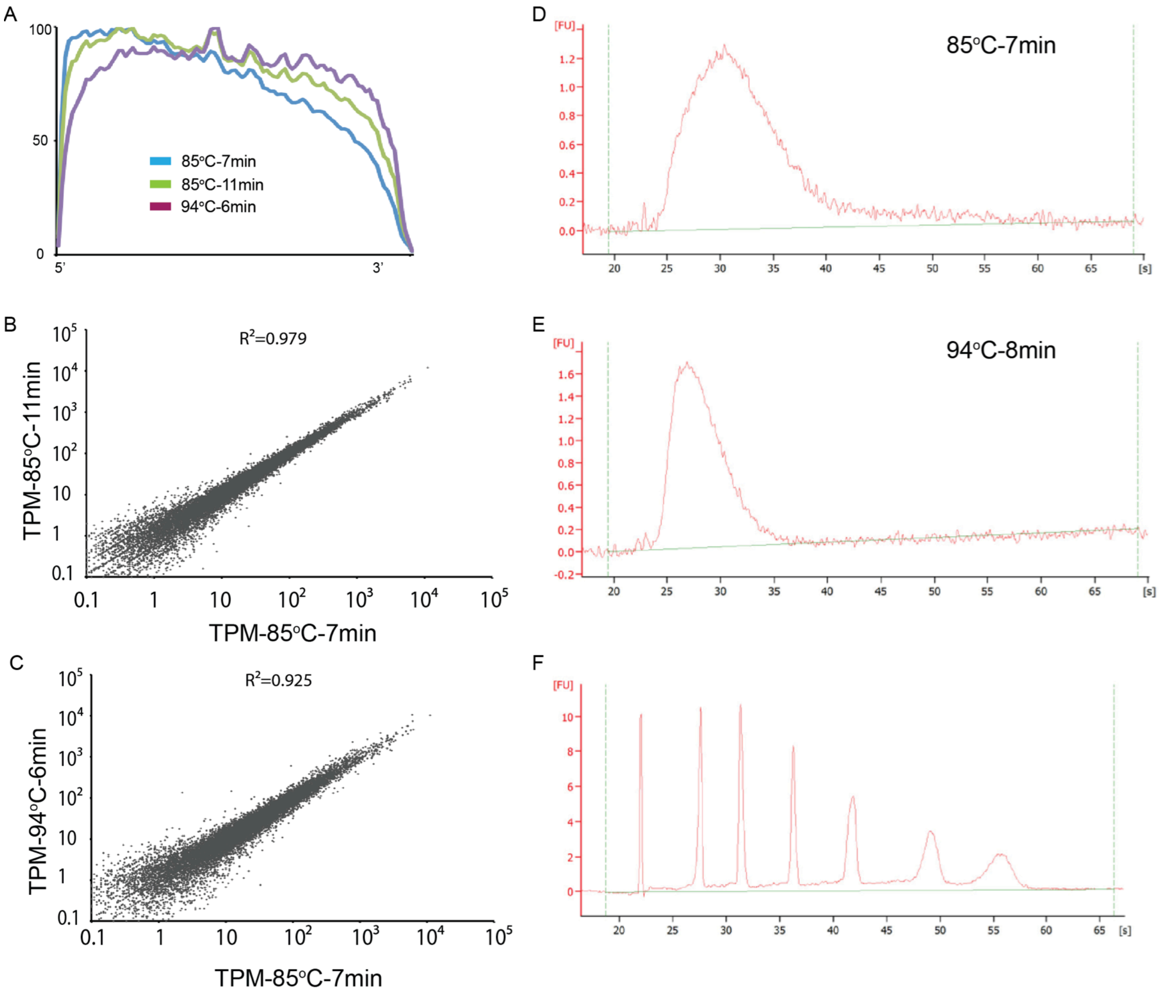

Supplemental Figure 1: Effect of RNA fragmentation on transcript read coverage. A. Average LM-Seq reads distribution across all transcripts under different RNA fragmentation conditions. Blue: 85°C for 7min. Green: 85°C for 11min. Purple: 94°C for 6min. B. and C. Scatter plot of TPM values of the same hES cells sample sequenced with LM-Seq using different RNA fragmentation conditions. Pearson correlation is reported on top of the plot. D. and E. Bioanalyzer electropherograms of fragmented mRNA using different fragmentation conditions. F. Bioanalyzer electropherogram of the RNA ladder.

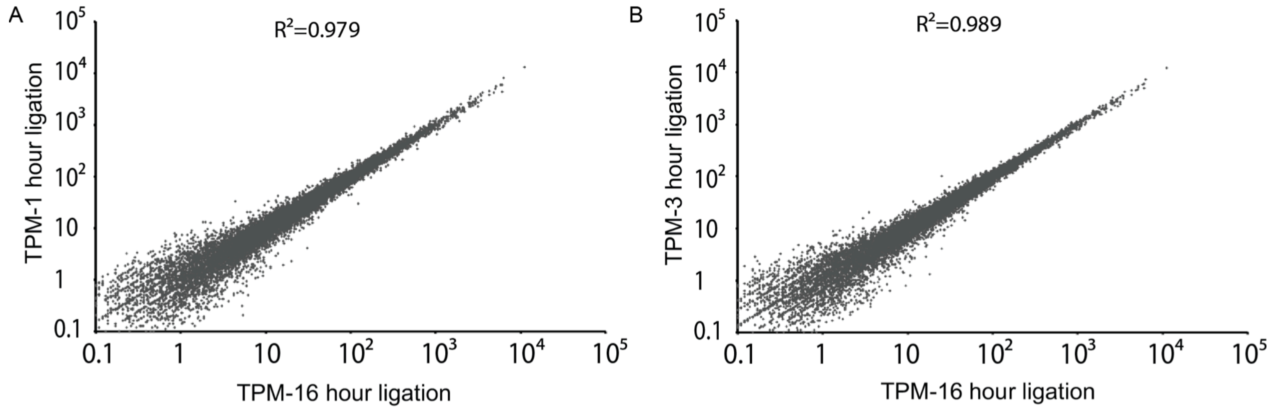

Supplemental Figure 2: Comparison of oligo ligation time. A. Scatter plot of TPM values of the same hES cells sample sequenced with LM-Seq using either 1hr or 16hr oligo ligation time. Pearson correlation is reported on top of the plot. B. Scatter plot of TPM values of the same hES cells sample sequenced with LM-Seq using either 3hr or 16hr oligo ligation time. Pearson correlation is reported on top of the plot.

Supplemental Table 1: Summary of reagent cost

Item	Vendor	Cat number	Price	Number of reactions	Cost per sample
NEBNext® Poly(A) mRNA Magnetic Isolation Module	NEB	E7490L	193.60	96.00	2.02
RiboGreen	Life Tech	R11490	429.00	1000.00	0.04
Qubit	Life Tech	Q32852	70.00	100.00	0.70
RNA 6000 Pico Kit	Agilent	5067-1513	672.00	275.00	0.22
3' AD HZG883	IDT	-	39.00	243.00	0.12
3' AD HZG885	IDT	-	39.00	243.00	0.24
5'AD LC056	IDT	-	39.00	243.00	0.08
ZH AD LIG 96	IDT	-	39.00	243.00	0.08
Ampure Beads	Agencourt	A63882	5493.00	450.00	2.51
RNase A	Epicentre	MRNA092	56.00	2000.00	0.00
RNase H	NEB	M0297L	256.00	625.00	0.41
10mM dNTP	Life Tech	18427088.00	399.00	1000.00	0.40
Nuclease Free Water	Life Tech	AM9932	60.00	1000000.00	0.00
RNase Inhibitor	Life Tech	10977-015	24.00	500.00	0.05
Ethanol (80%)	Sigma	E7023-6X500ML	279.00	100000.00	0.00
EB Buffer	Qiagen	19086.00	31.10	250.00	0.01
T4 RNA Ligase 1	NEB	M0204L	201.60	500.00	1.21
Smartscribe Rev Trans	Clontech	639538.00	550.00	400.00	1.38
FailSafe PCR Enzyme	Epicentre	FS9901K	1013.00	400.00	1.52
FailSafe PCR - Premix E	Epicentre	FSP995E-INCL	0.00	2500.00	0.00
PicoGreen	Life Tech	P7589	379.00	1000.00	0.04
1.5mL Tubes	Life Tech	AM12450	42.75	500.00	0.09
PCR Tubes	Life Tech	AM12230	153.00	125.00	1.22
p20 tips	Rainin	RT-L10F	45.93	9600.00	0.19
p200 tips	Rainin	RT-L200F	91.85	9600.00	0.53
p1000 tips	Rainin	RT-L1000F	91.85	7680.00	0.26
Total per Sample					13.32

Supplemental Table 2: Index primer sequences*

Primer Name	Reverse complement of the index embedded in the FNC of the adapter
ZH001	CAAGCAGAAGACGGCATACGAGATttggttaggtGTGACTGGAGTTCCTTGGCACCCGAGAATTCCA
ZH002	CAAGCAGAAGACGGCATACGAGATttggaattaaGTGACTGGAGTTCCTTGGCACCCGAGAATTCCA
ZH003	CAAGCAGAAGACGGCATACGAGATttgataggcaGTGACTGGAGTTCCTTGGCACCCGAGAATTCCA
ZH004	CAAGCAGAAGACGGCATACGAGATttgaagactcGTGACTGGAGTTCCTTGGCACCCGAGAATTCCA
ZH005	CAAGCAGAAGACGGCATACGAGATttctcttccgGTGACTGGAGTTCCTTGGCACCCGAGAATTCCA
ZH006	CAAGCAGAAGACGGCATACGAGATttcgtcgctaGTGACTGGAGTTCCTTGGCACCCGAGAATTCCA
ZH007	CAAGCAGAAGACGGCATACGAGATttcggctgagGTGACTGGAGTTCCTTGGCACCCGAGAATTCCA
ZH008	CAAGCAGAAGACGGCATACGAGATttcataacggGTGACTGGAGTTCCTTGGCACCCGAGAATTCCA
ZH009	CAAGCAGAAGACGGCATACGAGATttcaatcgttGTGACTGGAGTTCCTTGGCACCCGAGAATTCCA
ZH010	CAAGCAGAAGACGGCATACGAGATttcaaggacgGTGACTGGAGTTCCTTGGCACCCGAGAATTCCA
ZH011	CAAGCAGAAGACGGCATACGAGATttagccggccGTGACTGGAGTTCCTTGGCACCCGAGAATTCCA
ZH012	CAAGCAGAAGACGGCATACGAGATtggaagctagGTGACTGGAGTTCCTTGGCACCCGAGAATTCCA
ZH013	CAAGCAGAAGACGGCATACGAGATtgcgcggtaaGTGACTGGAGTTCCTTGGCACCCGAGAATTCCA
ZH014	CAAGCAGAAGACGGCATACGAGATtgatgaagcgGTGACTGGAGTTCCTTGGCACCCGAGAATTCCA
ZH015	CAAGCAGAAGACGGCATACGAGATtgaatgcgccGTGACTGGAGTTCCTTGGCACCCGAGAATTCCA
ZH016	CAAGCAGAAGACGGCATACGAGATtcttgacctcGTGACTGGAGTTCCTTGGCACCCGAGAATTCCA
ZH017	CAAGCAGAAGACGGCATACGAGATtcttacggtaGTGACTGGAGTTCCTTGGCACCCGAGAATTCCA
ZH018	CAAGCAGAAGACGGCATACGAGATtctgagaaggGTGACTGGAGTTCCTTGGCACCCGAGAATTCCA
ZH019	CAAGCAGAAGACGGCATACGAGATtctccgccagGTGACTGGAGTTCCTTGGCACCCGAGAATTCCA
ZH020	CAAGCAGAAGACGGCATACGAGATtctaggttgcGTGACTGGAGTTCCTTGGCACCCGAGAATTCCA
ZH021	CAAGCAGAAGACGGCATACGAGATtctaattgcgGTGACTGGAGTTCCTTGGCACCCGAGAATTCCA
ZH022	CAAGCAGAAGACGGCATACGAGATtcattcctcgGTGACTGGAGTTCCTTGGCACCCGAGAATTCCA
ZH023	CAAGCAGAAGACGGCATACGAGATtcaagcgaggGTGACTGGAGTTCCTTGGCACCCGAGAATTCCA
ZH024	CAAGCAGAAGACGGCATACGAGATtaggcttatgGTGACTGGAGTTCCTTGGCACCCGAGAATTCCA
ZH025	CAAGCAGAAGACGGCATACGAGATtacgtaggacGTGACTGGAGTTCCTTGGCACCCGAGAATTCCA
ZH026	CAAGCAGAAGACGGCATACGAGATtaacgagattGTGACTGGAGTTCCTTGGCACCCGAGAATTCCA
ZH027	CAAGCAGAAGACGGCATACGAGATgttcggcatgGTGACTGGAGTTCCTTGGCACCCGAGAATTCCA
ZH028[&]	CAAGCAGAAGACGGCATACGAGATgttaggagccGTGACTGGAGTTCCTTGGCACCCGAGAATTCCA
ZH029	CAAGCAGAAGACGGCATACGAGATgttaatggaaGTGACTGGAGTTCCTTGGCACCCGAGAATTCCA
ZH030	CAAGCAGAAGACGGCATACGAGATgtcaactctgGTGACTGGAGTTCCTTGGCACCCGAGAATTCCA
ZH031	CAAGCAGAAGACGGCATACGAGATgtagtattgcGTGACTGGAGTTCCTTGGCACCCGAGAATTCCA
ZH032	CAAGCAGAAGACGGCATACGAGATgtaggtactgGTGACTGGAGTTCCTTGGCACCCGAGAATTCCA
ZH033	CAAGCAGAAGACGGCATACGAGATgtactggtaaGTGACTGGAGTTCCTTGGCACCCGAGAATTCCA
ZH034	CAAGCAGAAGACGGCATACGAGATggttcatctgGTGACTGGAGTTCCTTGGCACCCGAGAATTCCA
ZH035	CAAGCAGAAGACGGCATACGAGATggtaagtatcGTGACTGGAGTTCCTTGGCACCCGAGAATTCCA
ZH036	CAAGCAGAAGACGGCATACGAGATggcggcaattGTGACTGGAGTTCCTTGGCACCCGAGAATTCCA

ZH037	CAAGCAGAAGACGGCATACGAGATggccaagtagGTGACTGGAGTTCCTTGGCACCCGAGAATTCCA
ZH038	CAAGCAGAAGACGGCATACGAGATggcattgatgGTGACTGGAGTTCCTTGGCACCCGAGAATTCCA
ZH039	CAAGCAGAAGACGGCATACGAGATggaataccagGTGACTGGAGTTCCTTGGCACCCGAGAATTCCA
ZH040	CAAGCAGAAGACGGCATACGAGATggaactaggcGTGACTGGAGTTCCTTGGCACCCGAGAATTCCA
ZH041	CAAGCAGAAGACGGCATACGAGATgcttgcctaaGTGACTGGAGTTCCTTGGCACCCGAGAATTCCA
ZH042	CAAGCAGAAGACGGCATACGAGATgctgcaaggaGTGACTGGAGTTCCTTGGCACCCGAGAATTCCA
ZH043	CAAGCAGAAGACGGCATACGAGATgctcttcgtaGTGACTGGAGTTCCTTGGCACCCGAGAATTCCA
ZH044	CAAGCAGAAGACGGCATACGAGATgctatctgacGTGACTGGAGTTCCTTGGCACCCGAGAATTCCA
ZH045	CAAGCAGAAGACGGCATACGAGATgctagagccgGTGACTGGAGTTCCTTGGCACCCGAGAATTCCA
ZH046	CAAGCAGAAGACGGCATACGAGATgcgtagagacGTGACTGGAGTTCCTTGGCACCCGAGAATTCCA
ZH047	CAAGCAGAAGACGGCATACGAGATgcggattcctGTGACTGGAGTTCCTTGGCACCCGAGAATTCCA
ZH048	CAAGCAGAAGACGGCATACGAGATgcgctgactgGTGACTGGAGTTCCTTGGCACCCGAGAATTCCA
ZH049	CAAGCAGAAGACGGCATACGAGATgcctgaatggGTGACTGGAGTTCCTTGGCACCCGAGAATTCCA
ZH050	CAAGCAGAAGACGGCATACGAGATgcagaggttgGTGACTGGAGTTCCTTGGCACCCGAGAATTCCA
ZH051	CAAGCAGAAGACGGCATACGAGATgcaataagttGTGACTGGAGTTCCTTGGCACCCGAGAATTCCA
ZH052	CAAGCAGAAGACGGCATACGAGATgcaacttaagGTGACTGGAGTTCCTTGGCACCCGAGAATTCCA
ZH053	CAAGCAGAAGACGGCATACGAGATgcaacgcctcGTGACTGGAGTTCCTTGGCACCCGAGAATTCCA
ZH054	CAAGCAGAAGACGGCATACGAGATgatggcggtcGTGACTGGAGTTCCTTGGCACCCGAGAATTCCA
ZH055	CAAGCAGAAGACGGCATACGAGATgaggagccgcGTGACTGGAGTTCCTTGGCACCCGAGAATTCCA
ZH056	CAAGCAGAAGACGGCATACGAGATgagccgcgcgGTGACTGGAGTTCCTTGGCACCCGAGAATTCCA
ZH057	CAAGCAGAAGACGGCATACGAGATgagcatggttGTGACTGGAGTTCCTTGGCACCCGAGAATTCCA
ZH058	CAAGCAGAAGACGGCATACGAGATgacttgattcGTGACTGGAGTTCCTTGGCACCCGAGAATTCCA
ZH059	CAAGCAGAAGACGGCATACGAGATgactaaggcaGTGACTGGAGTTCCTTGGCACCCGAGAATTCCA
ZH060	CAAGCAGAAGACGGCATACGAGATcttacgtcggGTGACTGGAGTTCCTTGGCACCCGAGAATTCCA
ZH061	CAAGCAGAAGACGGCATACGAGATctcttcaaccGTGACTGGAGTTCCTTGGCACCCGAGAATTCCA
ZH062	CAAGCAGAAGACGGCATACGAGATctcgctggatGTGACTGGAGTTCCTTGGCACCCGAGAATTCCA
ZH063	CAAGCAGAAGACGGCATACGAGATctcgcatctcGTGACTGGAGTTCCTTGGCACCCGAGAATTCCA
ZH064	CAAGCAGAAGACGGCATACGAGATctcaacctgcGTGACTGGAGTTCCTTGGCACCCGAGAATTCCA
ZH065	CAAGCAGAAGACGGCATACGAGATctagacgcagGTGACTGGAGTTCCTTGGCACCCGAGAATTCCA
ZH066	CAAGCAGAAGACGGCATACGAGATctaccagccaGTGACTGGAGTTCCTTGGCACCCGAGAATTCCA
ZH067	CAAGCAGAAGACGGCATACGAGATcgtagcaagcGTGACTGGAGTTCCTTGGCACCCGAGAATTCCA
ZH068	CAAGCAGAAGACGGCATACGAGATcgcatcttcgGTGACTGGAGTTCCTTGGCACCCGAGAATTCCA
ZH069	CAAGCAGAAGACGGCATACGAGATcgagaatatgGTGACTGGAGTTCCTTGGCACCCGAGAATTCCA
ZH070	CAAGCAGAAGACGGCATACGAGATcctgctattgGTGACTGGAGTTCCTTGGCACCCGAGAATTCCA
ZH071	CAAGCAGAAGACGGCATACGAGATccatataacgGTGACTGGAGTTCCTTGGCACCCGAGAATTCCA
ZH072	CAAGCAGAAGACGGCATACGAGATccaatgatacGTGACTGGAGTTCCTTGGCACCCGAGAATTCCA
ZH073	CAAGCAGAAGACGGCATACGAGATattgatgctcGTGACTGGAGTTCCTTGGCACCCGAGAATTCCA
ZH074	CAAGCAGAAGACGGCATACGAGATatggtaattgGTGACTGGAGTTCCTTGGCACCCGAGAATTCCA
ZH075	CAAGCAGAAGACGGCATACGAGATatgcgaccggGTGACTGGAGTTCCTTGGCACCCGAGAATTCCA

ZH076	CAAGCAGAAGACGGCATACGAGATatgcctgataGTGACTGGAGTTCCTTGGCACCCGAGAATTCCA
ZH077	CAAGCAGAAGACGGCATACGAGATatcttggcgcGTGACTGGAGTTCCTTGGCACCCGAGAATTCCA
ZH078	CAAGCAGAAGACGGCATACGAGATatatagcctgGTGACTGGAGTTCCTTGGCACCCGAGAATTCCA
ZH079	CAAGCAGAAGACGGCATACGAGATataggctatcGTGACTGGAGTTCCTTGGCACCCGAGAATTCCA
ZH080	CAAGCAGAAGACGGCATACGAGATataattagcgGTGACTGGAGTTCCTTGGCACCCGAGAATTCCA
ZH081	CAAGCAGAAGACGGCATACGAGATagttgctgccGTGACTGGAGTTCCTTGGCACCCGAGAATTCCA
ZH082	CAAGCAGAAGACGGCATACGAGATagttccgtagGTGACTGGAGTTCCTTGGCACCCGAGAATTCCA
ZH083	CAAGCAGAAGACGGCATACGAGATaggaatgaccGTGACTGGAGTTCCTTGGCACCCGAGAATTCCA
ZH084	CAAGCAGAAGACGGCATACGAGATagcttctcttGTGACTGGAGTTCCTTGGCACCCGAGAATTCCA
ZH085	CAAGCAGAAGACGGCATACGAGATagactggccgGTGACTGGAGTTCCTTGGCACCCGAGAATTCCA
ZH086	CAAGCAGAAGACGGCATACGAGATagacgtagatGTGACTGGAGTTCCTTGGCACCCGAGAATTCCA
ZH087	CAAGCAGAAGACGGCATACGAGATacttatctgcGTGACTGGAGTTCCTTGGCACCCGAGAATTCCA
ZH088	CAAGCAGAAGACGGCATACGAGATactggtaaccGTGACTGGAGTTCCTTGGCACCCGAGAATTCCA
ZH089	CAAGCAGAAGACGGCATACGAGATactgcattacGTGACTGGAGTTCCTTGGCACCCGAGAATTCCA
ZH090	CAAGCAGAAGACGGCATACGAGATactctagatgGTGACTGGAGTTCCTTGGCACCCGAGAATTCCA
ZH091	CAAGCAGAAGACGGCATACGAGATacggatggagGTGACTGGAGTTCCTTGGCACCCGAGAATTCCA
ZH092	CAAGCAGAAGACGGCATACGAGATacgcgcaagtGTGACTGGAGTTCCTTGGCACCCGAGAATTCCA
ZH093	CAAGCAGAAGACGGCATACGAGATacgacgcgaaGTGACTGGAGTTCCTTGGCACCCGAGAATTCCA
ZH094	CAAGCAGAAGACGGCATACGAGATacctctccatGTGACTGGAGTTCCTTGGCACCCGAGAATTCCA
ZH095	CAAGCAGAAGACGGCATACGAGATaccagcagcaGTGACTGGAGTTCCTTGGCACCCGAGAATTCCA
ZH096	CAAGCAGAAGACGGCATACGAGATaattggctcgGTGACTGGAGTTCCTTGGCACCCGAGAATTCCA

*: Oligonucleotide sequences © 2007-2013 Illumina, Inc. All rights reserved. Derivative works created by Illumina customers are authorized for use with Illumina instruments and products only. All other uses are strictly prohibited.

&: This index was not included in the test sample sets reported in the manuscript due to failed library amplification, likely due to accidental sample loss during purification. However, it has been used in other library preparations using LM-Seq with very good results.
